# Xenotransplantation and interspecies organogenesis: current status and issues

**DOI:** 10.3389/fendo.2022.963282

**Published:** 2022-08-05

**Authors:** Mayuko Kano, Eiji Mizutani, Shota Homma, Hideki Masaki, Hiromitsu Nakauchi

**Affiliations:** ^1^ Stem Cell Therapy Laboratory, Advanced Research Institute, Tokyo Medical and Dental University, Tokyo, Japan; ^2^ Laboratory of Stem Cell Therapy, Faculty of Medicine, University of Tsukuba, Tsukuba, Japan; ^3^ Institute for Stem Cell Biology and Regenerative Medicine, Stanford University School of Medicine, Stanford, CA, United States

**Keywords:** xenotransplantation, blastocyst complementation, pluripotent stem cells, chimera, type 1 diabetes, pancreas, islet

## Abstract

Pancreas (and islet) transplantation is the only curative treatment for type 1 diabetes patients whose β-cell functions have been abolished. However, the lack of donor organs has been the major hurdle to save a large number of patients. Therefore, transplantation of animal organs is expected to be an alternative method to solve the serious shortage of donor organs. More recently, a method to generate organs from pluripotent stem cells inside the body of other species has been developed. This interspecies organ generation using blastocyst complementation (BC) is expected to be the next-generation regenerative medicine. Here, we describe the recent advances and future prospects for these two approaches.

## Introduction

Type 1 diabetes mellitus and diabetes after total pancreatectomy completely deplete insulin secretion. Patients with insulin-dependent diabetes administrate exogenous insulin to control blood glucose levels. However, depletion of a patient’s own insulin secretion makes blood glucose levels extremely unstable, which results not only in the progression of diabetic complications but also in life-threatening and life-long deterioration of quality of life due to severe hypoglycemia. Pancreas transplantation is a great hope for patients suffering from insulin-dependent diabetes because it is the only curative treatment available today. Successful pancreas transplantation can lead to normalization of blood glucose and HbA1c levels which allow patients to be free from daily insulin injections (1). Pancreas transplantation is considered a good option because the glucagon response to hypoglycemia can be restored simultaneously, preventing prolonged hypoglycemia (2). Furthermore, pancreas transplantation may be superior to conventional insulin therapy from the standpoint of health economics (3). Currently, the majority of pancreases as transplant organs rely on donations from brain-dead donors. However, many patients are unable to receive pancreas transplantation due to donor organ shortage. Instead of human-to-human allogeneic transplantation, animal-derived organ transplantation (xenotransplantation) has been expected to solve organ shortage. In January 2022, the University of Maryland School of Medicine announced that they had successfully transplanted a genetically modified pig heart into a patient with end-stage cardiac disease. Unfortunately, the patient passed away about 2 months after the transplantation, but it made a big step toward the clinical application of xenotransplantation.

## Hurdles standing in xenotransplantation

Xenotransplantation has a long history, with its initial concept of transplanting part of the pancreas or its extract from a heterologous animal into a diabetic patient, first reported more than 100 years ago; in 1894, Williams et al. transplanted minced sheep pancreas subcutaneously into a type 1 diabetic boy with ketoacidosis (4). At that time, xenotransplantation was a reckless challenge because effective immunosuppressive agents and gene editing techniques were not developed yet. In the early days, non-human primate (NHP) organ transplantation was widely studied (5). However, smaller organ size, ethical concerns, the risk of cross-species transmission between humans and NHPs, and the difficulty of reproduction led to a gradual decline. Next, pigs became a candidate organ donor for the similarity in organ size and physiology and for their reproductivity. Despite those advantages, there were remaining difficulties for clinical application, which are immune rejection and pig-to-human infection.

## Overcoming hyperacute rejection

What happens when wild-type porcine organs are transplanted into humans? The human immune system recognizes the antigens expressed on the surface of porcine cells and starts attacking immediately after transplantation and results in hyperacute rejection (HAR). It progresses from a few minutes to several tens of minutes, until it gets to the point that the graft loses its function completely. Galactose-a1,3-galactose (α-Gal) was first noticed as the major xenograft antigen in pig-to-human transplantation (6). α-Gal is present in many mammals, including pigs, but not in some primates such as humans and Old-World monkeys. In these primates, natural antibodies against α-Gal exist in blood (7). When anti-α-Gal antibodies bind to the α-Gal antigen expressed on the surface of porcine endothelial cells, which induces the activation of complement proteins, the graft is eliminated (8). To avoid HAR, the gene-encoding α1,3-galactosyltransferase (α1,3GalT, GGTA1) knockout (KO) pigs were required. In 2001, α1,3GalT heterozygous KO pigs were developed at PPL Therapeutics (now Revivicor) and Immerge BioTherapeutics (9, 10) *via* cloning by somatic cell nuclear transfer. The following year, α1,3GalT was homozygously knocked out, and cloned pigs with the α-Gal antigen completely eliminated were generated (11, 12). To date, two other xenoantigens that were problematic for transplantation, namely, N-glycolylneuraminic acid (Neu5Gc) (13) and β1,4-N-acetylgalactosyltransferase (β4GalNT2, SDa) (14), were defined. Attempts have been made to overcome these new xenoantigens by developing new transgenic pigs (15–17). The triple KO (TKO) pigs, which eliminate α-Gal, Neu5Gc, and the SDa epitopes, exhibited reduced human antibody binding *in vitro* (18). Thus, TKO pigs became the backbone of any genetically engineered pig.

## Preventing pig-to-human infection

The second barrier is the presence of porcine endogenous retroviruses (PERVs), which are carried in almost all pigs. PERVs are harmless to pigs; however, potential pathogenicity to humans remains unclear. PERVs have been observed to be transmitted from pig to human and human to human *in vitro* (19). Since organ transplant recipients are under strong immunosuppression, it is desirable to avoid PERV infection. To this point, the generation of PERV-free pigs has been considered. It was considered impossible to inactivate all of the PERV genes before since PERV is integrated into multiple sites of the porcine genome. The median copy number of PERVs ranged between 46 and 70 copies, although some were found to have more than 100 copies (20). The CRISPR–Cas9 technology (21) enabled us to overcome this. Yang et al. inactivated all copies of the PERV gene from porcine somatic cells using CRISPR–Cas9 (22). They also generated PERV-free pigs from PERV gene-inactivated porcine somatic cells using somatic cell nuclear transfer (23). Xenotransplantation can be free from PERV infection risk by using PERV-free pigs as a donor.

## Advances in transgenic pigs as organ donors

To prevent xenograft rejection, new genetically engineered pigs have been developed based on GalT-KO pigs. While HAR to xenoantigens was humoral-mediated immunity triggered by already existing natural antibodies, cellular immunity is also involved in xenograft rejection. Human NK cells are considered to play an important role in cellular xenograft rejection (24). They are capable of lysing cells with reduced human major histocompatibility complex (MHC) class I expression. High expression of HLA-E, one of the MHC class I molecules, allows porcine cells to evade human NK cytotoxicity (25). Human CD47 expression in porcine cells prevents phagocytosis from human macrophages (26). To regulate complement activation, overexpression of human complement regulatory proteins (CRPs) was attempted (27, 28). Apart from the immune system, coagulation dysregulation is also involved in xenograft rejection. Human coagulation regulatory proteins were expressed to prevent human platelet aggregation in the vessels of the xenografts (29, 30). Finally, porcine organ size was optimized. A rapid increase in graft size has been reported when porcine organs are transplanted into NHPs (31). To solve this problem, pigs suitable for transplantation were generated by knocking out the receptors for growth hormones (32). Revivicor developed genetically engineered pigs with 10 genetic modifications (10-GE pigs). The 10-GE pigs have targeted insertion of six human genes, consisting of two human complement inhibitor genes (*hDAF*, *hCD46*), two human anticoagulant genes (*hTBM*, *hEPCR*), and two immunomodulatory genes (*hCD47*, *hHO1*), as well as KO of three porcine xenoantigens (α-Gal, Neu5Gc, and SDa) and the porcine growth hormone receptor gene. It became possible to generate pigs with multiple gene modifications by gene editing and nuclear transfer, thereby overcoming the various problems that hampered xenotransplantation.

## Toward clinical application

As a result of further development of genetically engineered pigs, many pig-to-NHP transplantations were performed as the final step toward preclinical studies. Among them, a pig-to-baboon heart transplant experiment at the U.S. National Institutes of Health (NIH) reported a long survival time of up to 945 days (33). In 2020, the U.S. Food and Drug Administration (FDA) approved the GalT-KO pigs, the platforms for all transgenic pigs, as a source of human therapeutics including xenotransplantation. In 2022, the University of Alabama reported that a kidney derived from a genetically modified pig was transplanted into a brain-dead decedent (34), which marked the first clinical application of xenotransplantation from a pig to a human. The 10-GE pig kidneys were transplanted into a man who was brain dead from head trauma after bilateral nephrectomy. No HAR and PERV transmission were observed. Although the porcine kidney produced urine within 30 min, serum creatinine did not decrease during the study period. Histological findings on postoperative periods demonstrated endothelial injury with diffuse thrombotic microangiopathy. The decedent’s general condition deteriorated after transplantation, leading to progressive multiple organ failure. Thus, the study was terminated only after 3 days. Further study, including the mechanisms and prevention of microvascular injuries, is required to uncover the etiology of poor renal recovery.

Pig islet transplantation into a human has a longer history than organ transplantation in xenotransplantation (35–37). Transplantation of encapsulated wild-type porcine islets to established brittle type 1 diabetes mellitus was performed without immunosuppression, which resulted in improved HbA1c and reduced unaware hypoglycemic events (37). However, only a slight decrease in insulin doses was observed, and no patient could achieve insulin independence. Aside from encapsulated islet transplantation, there have been attempts to transplant naked porcine islets into NHPs. Xenotransplantation of neonatal porcine islets from α1,3GalT KO piglets into immunosuppressed STZ-induced diabetic rhesus monkeys resulted in reduced complement-mediated rejection (38). Various genetically engineered pigs have been developed to protect pig islets from the host’s immune responses. However, there are still problems regarding effectiveness and long-term survival. Future studies will reveal whether the new genetically engineered pigs, including the 10-GE pigs, will solve these problems.

## Blastocyst complementation: Another approach for organ generation

**Figure 1:**
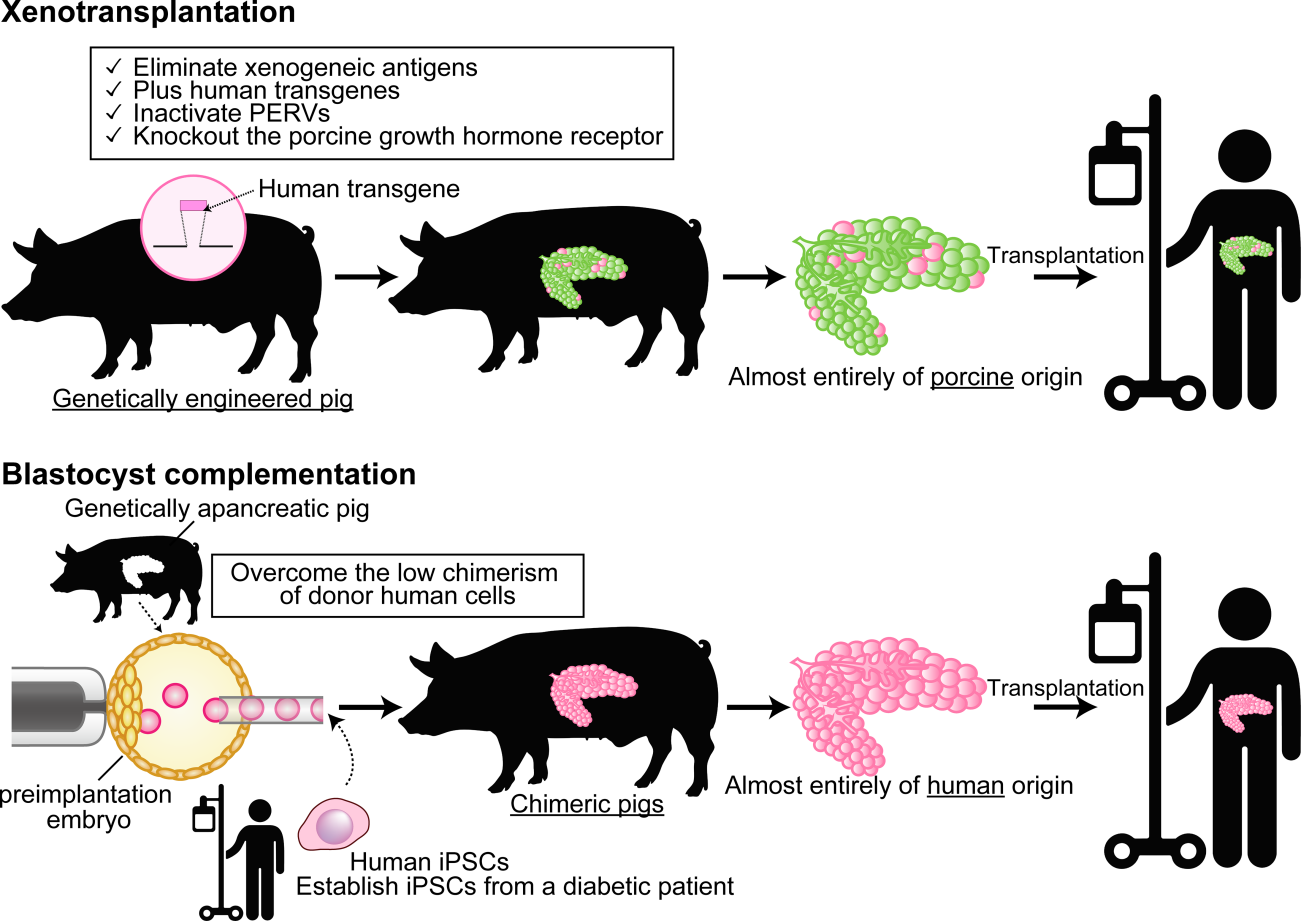
Differences between xenotransplantation and animal-developed human organ transplantation by using blastocyst complementation. In xenotransplantation (top), a small percentage of the xenografts have proteins derived from the human transgene, but the majority are of porcine origin. On the other hand, the blastocyst complementation (bottom) generates organs almost completely of human origin in a specific organ-deficient animal. Cartoon pictures (human transgene, pig, and human) were created with BioRender.

The other approach to solve organ shortage is by creating a human organ from human pluripotent stem cells (PSCs) in an animal body by using interspecies blastocyst complementation (BC). In this approach, the patient’s own induced PSCs (iPSCs) are injected into the targeted organ-deficient animal’s preimplantation embryos, and then the defective organs are completely replaced by donor cells when the embryo develops to full term. It means that, when the technology is realized for humans, a BC-derived organ is expected to be functional and rejection-free (39) (**Figure 1**).

The concept of BC was first reported in lymphocyte complementation by using *Rag2*-KO mouse embryos (40). Chen et al. indicated that B- and T-lymphocyte deficiencies in *Rag2* KO were fully covered by donor cells in wild-type mouse embryonic stem cell (mESC)-injected chimeras. They also complemented *Rag2* KO blastocysts with mESCs lacking immunoglobulin heavy-chain joining (*IGHJ*) gene segment, and the chimeras had normal T cells but no B cells. This result demonstrated that the *IGHJ* gene segment is essential for B-cell development. As described, Chen and colleagues proposed BC as a functional assay to clarify the definitive role of targeted genes in development.

**Figure 2:**
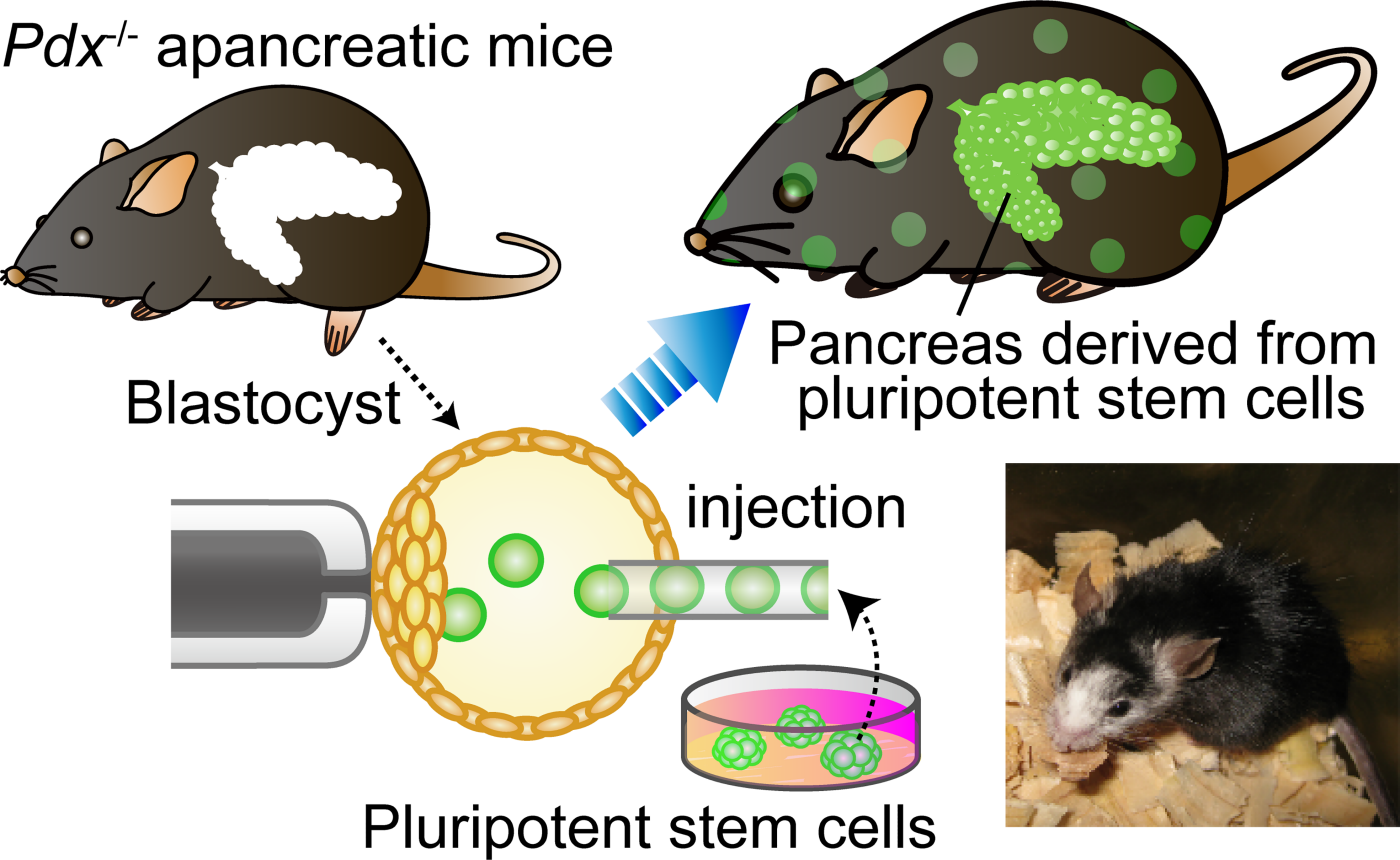
Blastocyst complementation using organ-deficient mice. Blastocyst complementation using *Pdx1* ^−/−^ pancreas-deficient mice. When wild-type pluripotent stem cells (PSCs) are injected into blastocysts derived from a genetically pancreas-deficient mouse, the injected PSCs can complement an empty developmental niche for a pancreas. Whereas the skin, tissues, and organs throughout the body of the chimeric mouse are composed of a mixture of PSCs and host embryos, the pancreas is composed entirely of cells derived from the donor PSCs. The lower right is a photograph of an interspecific chimera. This *Pdx1* ^−/−^ mouse complemented with rat PSCs has a pancreas derived from rat PSCs.

BC was firstly proposed as a technology to generate a whole organ with donor PSCs by Kobayashi etal. (41) (**Figure 2**). The first targeted organ was the pancreas because of the medical needs for pancreatic islet, and the apancreatic phenotype can be induced by the single-gene KO of *Pdx1* (pancreatic and duodenal homeobox1) (42). Not only in an allogeneic setting but also in a rat–mouse interspecies setting, the pancreas was entirely complemented with donor cells in *Pdx1* ^−/−^ mice. Furthermore, these chimeras derived from *Pdx1* ^−/−^ mouse embryos grew to adulthood, maintained normal blood glucose levels, and secreted insulin in response to glucose tolerance tests (GTTs). These results indicated that donor PSCs can compensate for a defective organ niche and form functional organs even in interspecies chimeras. A reciprocal experiment was then performed for islet transplantation by generating rat-sized mouse pancreas in *Pdx1* ^−/−^ rat-derived interspecies chimeras (43). Mouse PSC-derived pancreas in mouse–rat chimera can maintain blood glucose level and respond to high glucose in GTT, albeit with a slightly delayed response compared to wild-type rats. One hundred mouse islets were isolated from *Pdx1* ^−/−^ interspecies chimeric rats and transplanted into the kidney capsule of STZ-induced diabetic mice. Although immunosuppression was stopped 5 days after transplantation, diabetic mice that received mPSC-derived islets exhibited normal blood glucose levels and normal responses to GTTs for over 1 year without continued immunosuppression. Flow cytometric analysis revealed that a substantial proportion of rat-supporting tissues were contaminated with the graft; however, the host immune system specifically eliminated contaminated xenogeneic cells without inducing HAR or damaging mouse insulin-producing cells.

In addition to the pancreas, many other organs have been complemented *via* BC, such as the kidney (44, 45), thymus (46), blood and vascular endothelial cells (47), lungs and bronchi (48), and gametes (49). BC-derived organ generation can be applicable to pigs (50), which is a great advantage in terms of realization because pigs have similar-sized organs to humans and have multiple pregnancies.

These results demonstrate that most organs or tissues can be generated by interspecies BC, and they are functional and expected not to cause HAR for as long as the contaminated xenogeneic cell portion is relatively small.

## Challenges to applying blastocyst complementation to humans

After the success of interspecies chimera formation in rodents (41, 43, 51), the generation of human PSC-derived interspecies chimeras has been attempted to evaluate its developmental potency and assess the possibility of human organ derivation. However, the contribution of human cells in interspecies chimera was very low in all the studies, and none of them showed significant donor chimerism in neonates or adults (52–56). Donor rat cell chimerism was about 20%–25% in most of the cases of rat–mouse interspecies chimeras (41). Donor human cell chimerism in human–mouse interspecies chimeras was reported to be 0.1%–4.0% (57), while higher results have been reported for human–pig interspecies chimeras (58). Nevertheless, it must be noted that the efficiency of human–animal chimeras is still low. While rats and mice diverged about 20 million years ago, humans diverged from mice and pigs nearly 100 million years ago (59). Such evolutional divergence of developmental molecules and systems might have prevented human cell contribution to host animal development.

Developmental stage mismatch between the host animal embryo and the donor human PSC would be the other reason for the difficulty in forming human–animal chimeras. Mouse ESCs and human ESCs are both derived from the preimplantation epiblast, but human conventional ESCs (and iPSCs) carry characteristics more common to postimplantation epiblast-derived mouse EpiSCs (60, 61). PSCs that resembled to preimplantation epiblast are called “naive” PSCs, and postimplantation epiblast-like PSCs are called “primed” PSCs (62). Mouse EpiSCs do not form chimeras when injected into preimplantation blastocysts, but they do form chimeras when implanted into postimplantation gastrula (63). This indicates that a certain level of developmental stage matching is required for chimera formation. Recently, the establishment of human naive-like PSCs has been reported (56, 64, 65). Although injected human naive PSCs showed poor contribution to animal development (52, 56), they might show difference to the conventional human PSCs when injected into evolutionally more closer species embryos, such as NHPs (66).

The other approach is the prevention of apoptosis in donor human primed PSCs. Our group previously reported that developmental stage-mismatched cells cause apoptosis when injected into preimplantation embryos. By blocking apoptosis in those mismatched donor cells, even the stage-mismatched cells could contribute to chimeras (67). Recently, it was confirmed that apoptosis-resistant human primed PSCs survive in mouse fetus (54, 55). However, only a few numbers of human cells survived in the chimeric embryos, and their contribution to organogenesis has not been reported. To overcome too low chimerism of donor human cells for organ complementation, our group developed the procedure, which allows a gradual increase of donor cell chimerism in later developmental stages by deleting the Igf1 receptor (Igf1r) of host cells in chimeras (68). By using the Igf1r KO animal embryos as a host, the progenies of human PSCs might increase their chimerism in the animal body and then complement the targeted organ or tissues.

In contrast to the successful results within rodents, human organ generation using BC has not been achieved yet. Multiple factors may need to be considered in future studies of generating human–animal chimeras, including the selection of animal species, gene editing of embryos and human PSC, and culture conditions for human PSCs.

## Discussion

This article introduced two concepts as approaches to transplant organ creation: xenotransplantation and interspecies BC. With xenotransplantation of pig-derived organs, many efforts have been made to overcome its major problem with rejection. Hyperacute rejection has been avoided through the use of gene editing techniques, but it is still not certain whether long-term engraftment in the human body is possible. Furthermore, it is unclear whether pig organs will be able to perform the same physiological functions as human organs. Another concern is the adverse effects of strong immunosuppression of the patient to reduce rejection. On the other hand, interspecies chimera-derived human organ transplantation aims to generate organs derived from the patient’s own iPSCs, which are autologous transplants, so the rejection is expected to be none or mild. Thus, there would be no need for immunosuppression. It has been suggested that the differences in the insulin secretion capacity and insulin demands to food intake between humans and pigs may be a problem in pig islet transplantation (69). In this regard, islets made from human PSCs by BC would not have this problem. However, further study is necessary to address the issue of the difficulty of human PSCs to contribute to animal embryos in the early developmental stage. Contaminated host animal cells, such as blood vessels, might cause rejection after transplantation. We previously indicated that those contaminated xenogeneic cells were cleared by the recipient’s immune system in BC-derived pancreatic islet transplantation (43); however, this immune reaction could trigger more intense rejection in orthotropic whole-organ transplantation. To solve this problem, vascular endothelial cell complementation (47, 70) along with targeted organ complementation might be required.

After all, these two types of approaches are not contradictory but rather synergistic; for example, genetically modified animals designed for xenotransplantation could be useful as a host for interspecies chimera-derived human organ as they carried xenogeneic endothelial cells which are less immunogenic to the human immune system. Furthermore, the utilization of PERV-free pigs may provide the ideal host for clinical interspecies organogenesis to prevent PERV transmission from host pig cells. Whichever method is chosen, the future direction of transplantation medicine using animals is to allow for a safe and practical method of growing organs in donor animals as well as saving many patients suffering from irreversible organ failures, including insulin-dependent diabetes mellitus. Just as science has solved many obstacles in the past, we believe that future research developments will lead to the realization of animal-based transplantation medicine.

## Author contributions

MK, EM, and HN conceived the study. MK wrote the manuscript. EM, HM, and SH revised the manuscript. All authors contributed to the article and approved the submitted version.

## Funding

This work was supported by grants from the Centers for Clinical Application Research on Specific Disease/Organ (to HN) of the Research Center Network for Realization of Regenerative Medicine, funded by the Japan Agency for Medical Research and Development (AMED) under Grant Number JP22bm1004002; Grant-in-Aid for Early-Career Scientists (to MK), funded by the Japan Society for the Promotion of Science (JSPS) KAKENHI under Grant Number JP21K16337.

## Acknowledgments

We thank Dr. M. Watanabe for critical advice in preparing the manuscript and K. Okada for secretarial support. The authors used BioRender in the creation of the figure.

## Conflict of interest

HN is a cofounder and shareholder in ReproCELL, Megakaryon, and Century Therapeutics.

The remaining authors declare that the research was conducted in the absence of any commercial or financial relationships that could be construed as a potential conflict of interest.

## Publisher’s note

All claims expressed in this article are solely those of the authors and do not necessarily represent those of their affiliated organizations, or those of the publisher, the editors and the reviewers. Any product that may be evaluated in this article, or claim that may be made by its manufacturer, is not guaranteed or endorsed by the publisher.
